# Non-surgical treatment before hip and knee arthroplasty remains underutilized with low satisfaction regarding performance of work, sports, and leisure activities

**DOI:** 10.1080/17453674.2020.1813440

**Published:** 2020-09-03

**Authors:** Yvonne Van Zaanen, Alexander Hoorntje, Koen L M Koenraadt, Leti Van Bodegom-Vos, Gino M M J Kerkhoffs, Suzanne Waterval-Witjes, Tim A E J Boymans, Rutger C I Van Geenen, P Paul F M Kuijer

**Affiliations:** a Department of Public and Occupational Health, Amsterdam UMC, University of Amsterdam, Amsterdam Public Health Research Institute, Amsterdam Movement Sciences, Amsterdam; b Department of Orthopaedic Surgery, Amphia Hospital, Foundation FORCE (Foundation for Orthopaedic Research Care and Education), Breda; c Department of Orthopaedic Surgery, Amsterdam UMC, University of Amsterdam, Academic Center for Evidence based Sports medicine (ACES), Amsterdam Movement Sciences, Amsterdam; d Department of Biomedical Data Sciences, Leiden University Medical Center, Leiden; e Personalized Knee Care, Maastricht; f Department of Orthopaedic surgery, Maastricht University Medical Center, Maastricht, The Netherlands

## Abstract

Background and purpose — Guidelines for managing hip and knee osteoarthritis (OA) advise extensive non-surgical treatment prior to surgery. We evaluated what percentage of hip and knee OA patients received non-surgical treatment prior to arthroplasty, and assessed patient satisfaction regarding alleviation of symptoms and performance of activities.

Patients and methods — A multi-center cross-sectional study was performed in 2018 among 186 patients who were listed for hip or knee arthroplasty or had undergone surgery within the previous 6 months in the Netherlands. Questions concerned non-surgical treatments received according to the Stepped Care Strategy and were compared with utilization in 2013. Additionally, satisfaction with treatment effects for pain, swelling, stiffness, and activities of daily life, work, and sports/leisure was questioned.

Results — The questionnaire was completed by 175 patients, age 66 years (range 38–84), 57% female, BMI 29 (IQR 25–33). Step 1 treatments, such as acetaminophen and lifestyle advice, were received by 79% and 60% of patients. Step 2 treatments, like exercise-based therapy and diet therapy, were received by 66% and 19%. Step 3—intra-articular injection—was received by 47%. Non-surgical treatment utilization was lower than in 2013. Nearly all treatments showed more satisfied patients regarding pain relief and fewer regarding activities of work/sports/leisure. Hip and knee OA patients were mostly satisfied with NSAIDs for all outcomes, while exercise-based therapy was rated second best.

Interpretation — Despite international guideline recommendations, non-surgical treatment for hip and knee OA remains underutilized in the Netherlands. Of the patients referred for arthroplasty, more were satisfied with the effect of non-surgical treatment on pain than on work/sports/leisure participation.

Worldwide guidelines for managing osteoarthritis (OA) of the hip and knee advise extensive non-surgical treatment prior to surgery (Zhang et al. 2010, Smink et al. 2011, McAlindon et al. 2014). Non-surgical treatment is cost-effective and may lower the rapidly increasing OA-related healthcare expenditure by delaying or even replacing surgery (Berwick and Hackbarth 2012).

The global Choosing Wisely initiative aims to optimize healthcare usage and costs by advocating the use of proven but underused healthcare modalities, including preventive care (Berwick and Hackbarth 2012, Bernstein 2015). Regarding hip and knee OA, studies have found underuse of non-surgical treatments (Snijders et al. 2011, Hofstede et al. 2015). For example, 1 study showed that 81% of hip and knee OA patients did not receive all recommended non-surgical treatments (Snijders et al. 2011). In the Netherlands, a Stepped Care Strategy (SCS) was developed to stimulate the use of non-surgical treatment before hip and knee replacement (Smink et al. 2011). Moreover, providing adequate non-surgical treatment before hip and knee replacement was recommended by the Dutch Orthopedic Association for their Choosing Wisely Campaign (NOV 2015). Yet, the actual utilization of non-surgical treatment in hip and knee OA patients prior to arthroplasty in the Netherlands is described only by a cohort study from 2013 (Hofstede et al. 2015). Furthermore, no previous study has simultaneously assessed patient satisfaction with non-surgical treatments regarding their effect on symptoms like pain and swelling, and participation as in daily life and work. This is of importance given the increasing number of hip and knee OA patients who want to eliminate their pain and also wish to remain active in daily life, work, and sport/leisure (Kurtz et al. 2009, Otten et al. 2010, Culliford et al. 2015, Witjes et al. 2017). Given the impact of surgery on work participation, the effect of non-surgical treatment on work participation is also of interest (Kuijer et al. 2016, Stigmar et al. 2017).

Therefore, the main aim was to assess preoperative non-surgical treatment by hip and knee OA patients referred for arthroplasty in 2018, as well as compared with 2013, and their satisfaction regarding alleviation of symptoms and performing activities of daily living (ADL), work, and sports/leisure activities.

## Patients and methods

### Multi-center study

A multi-center cross-sectional online questionnaire study was performed by convenience sampling, similar to the Dutch study of 2013 (Hofstede et al. 2015), between October 2017 and April 2018 in 5 Dutch hospitals. Eligible patients were either listed for total hip arthroplasty (THA) or total knee arthroplasty (TKA) or had undergone THA or TKA less than 6 months previously in order to minimize recall bias. The participating hospitals were located in the northern, central, and southern parts of the Netherlands, including city and rural areas, and serving general THA and TKA populations. All patients received written information concerning the study and an online invitation to participate. If they agreed to participate, they received the invitation to fill out the online questionnaire. Furthermore, all participating patients received a gift card to the value of 10 euros after completing the questionnaire.

### Questionnaire

An online questionnaire was developed using an electronic data management system (Castor EDC, www.castoredc.com). Eligible patients received an invitation by email, followed by a maximum of 2 email reminders. The questionnaire started with questions regarding baseline characteristics, including age, sex, bodyweight and height, educational level, comorbidity, work situation, and onset of OA complaints.

### Non-surgical treatment modalities

Received non-surgical treatment modalities were asked about based on the multidisciplinary guideline, which consists of 3 sequenced steps, called the Stepped Care Strategy (SCS) (Smink et al. 2011). These 3 steps are: (1) education, lifestyle advice, acetaminophen, glucosamine sulfate (optional); (2) exercise-based therapy, diet therapy, NSAIDs, tramadol; and (3) intra-articular injection. Multidisciplinary care, a treatment option of step 3, was left out because this consists of treatment modalities similar to the monodisciplinary care of step 1 and 2 (Smink et al. 2011), which patients might find hard to distinguish.

To assess whether the Dutch SCS and Choosing Wisely campaign (2015) resulted in higher utilization of non-surgical therapy, our results were compared with a similar Dutch study performed in 2013 (Hofstede et al. 2015). Higher utilization was defined as a 10% higher utilization rate (Grimshaw et al. 2004).

### Patient satisfaction

Patients were asked to rate their satisfaction regarding the treatment effect of the above-mentioned non-surgical treatment modalities on a Likert scale from 1 (very unsatisfied) to 10 (very satisfied). For every received treatment 6 satisfaction rates were asked about for the effect on symptoms: pain, swelling, and stiffness; and on participation: daily life, work, and sports/leisure. These outcomes are in line with the OMERACT-OARSI core domain set (Singh et al. 2017, Smith et al. 2019). A cut-off point of 6 or higher was used to distinguish between “satisfied” and “not satisfied.”

### Statistics

Patient characteristics were described for the baseline characteristics such as age, sex, BMI, and onset of OA complaints. Subgroup analyses were performed for hip versus knee OA patients. Statistical differences in baseline characteristics and received treatments were tested between these subgroups of OA patients using a Student’s t-test, Mann–Whitney U-test, or Fisher–Freeman–Halton Exact test if corresponding assumptions were met. 2 sensitivity analyses were performed: (1) among patients who completed the questionnaire preoperatively versus postoperatively to explore whether this biased the results and (2) among patients in paid employment to assess their satisfaction with treatment effects for work participation to decide whether the results were biased by employment status at the time of surgery. All statistical analyses were performed using SPSS for Windows (Version 24.0; IBM Corp, Armonk, NY, USA). A significance level of p ≤ 0.05 was used.

### Ethics, funding, and potential conflicts of interest

The Medical Ethics Review Committee of the Amsterdam UMC, location Academic Medical Center, confirmed that the Medical Research Involving Human Subjects Act (WMO) did not apply to this study and official approval is not required (reference number W17_325 #17.378). Informed consent was obtained from all participants included in the study. This project received funding from the Netherlands Organisation for Health Research and Development (ZonMw) (reference number 516000503). The funder had no role in the conducting of the study or the decision to publish. No competing interests were declared.

## Results

**Figure 1.**
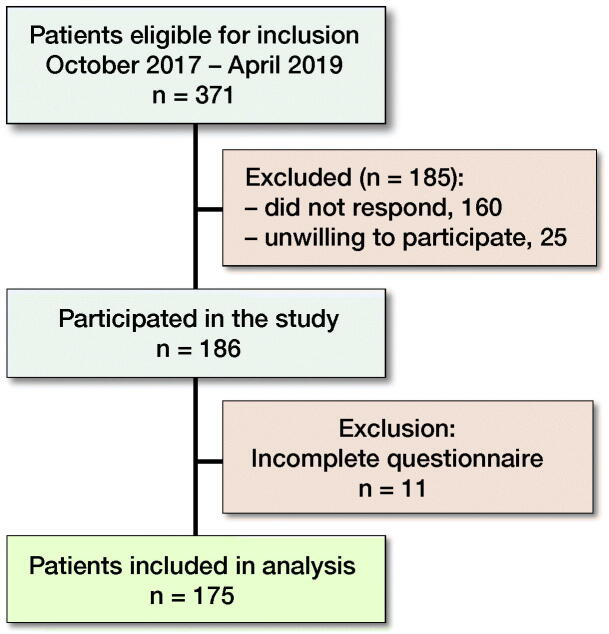
Flowchart of participating patients.

Of 371 invited patients, 186 were willing to participate (response rate 50%) and 175 patients completed the questionnaire (completion rate 94%; Figure 1). Mean age was 66 (SD 8) years, 57% were female, 3 out of 4 were overweight (median BMI 29 [IQR 25–33]) and 67% had no paid employment at the time of surgery (Table). Finally, in half of the patients, OA complaints lasted longer than 5 years.

### Differences between hip and knee OA patients

The median BMI of knee OA patients was statistically significantly higher (31 [IQR 27–36]) than that of the hip OA patients (28 [IQR 24–31]), and complaints lasted longer in knee OA patients (complaints > 5 years in 62%) than in hip OA patients (complaints > 5 years in 37%; Table).

### Received non-surgical treatments

**Figure 2.**
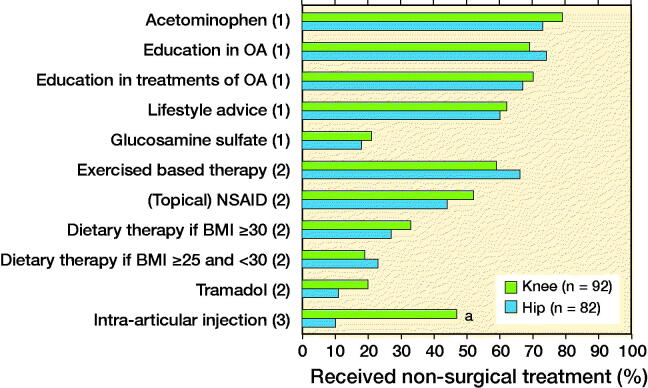
Non-surgical treatment received by hip and knee osteoarthritis (OA) patients according to the Stepped Care Strategy (Step 1, 2, or 3). ^a^ Significant difference between hip and knee OA patients (Fisher–Freeman–Halton Exact test p ≤ 0.05).

Of the SCS treatments of step 1, acetaminophen was received most often (hip 73%, knee 79%; p = 0.4) and lifestyle advice least often (hip 60%, knee 62%; p = 0.9) (Figure 2). Glucosamine sulfate was received by 18% of hip OA patients and 21% of knee OA patients (p = 0.7). Of the SCS treatments of step 2, exercise-based therapy was received most often by patients (hip 66%, knee 59%; p = 0.4), while diet therapy among overweight patients (hip 23%, knee 19%; p = 0.3) and tramadol (hip 11%, knee 20%; p = 0.1) were least often received. Intra-articular injections, the SCS step 3 treatment, were more often received by knee OA patients (47%) than by hip OA patients (10%; p < 0.01).

### Comparison of non-surgical treatment utilization over time

In comparison with the data from 2013 patients in 2018 were older (mean age 66 versus 64; p = 0.03), less often female (57% versus 72%; p = 0.01), more overweight (median 29 [IQR 25–33] versus 28 [25–31]), and their OA complaints lasted longer (> 5 years 50% versus 43%; p <0.01).

In 2018, the hip and knee OA patients reported having received less exercise-based therapy (–12%, prevalence ratio (PR) = 0.85 (95% confidence interval [CI] 0.73–0.99), less glucosamine sulfate (–16%, PR = 0.56 [CI 0.39–0.87]), fewer NSAIDs (–15%, PR = 0.76 [CI 0.63–0.92]), and less tramadol (–10%, PR = 0.62 [CI 0.40–0.95]) than in 2013. For the other non-surgical treatments, non-significant differences in utilization of less than 10% were found.

### Satisfaction with non-surgical treatment

**Figure 3.**
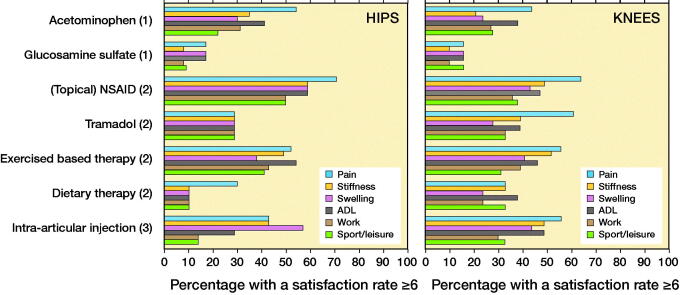
Percentage of hip and knee OA patients with a satisfaction rate ≥ 6 for effect of non-surgical treatment according to the Stepped Care Strategy (Step 1, 2, or 3) on pain, stiffness, swelling, activities of daily life (ADL), work, and sports/leisure.

Regarding acetaminophen, 54% of the hip OA patients were satisfied with the effect on pain, 35% on stiffness, 30% on swelling, 41% on ADL, 31% on work, and 22% on sport and leisure participation (Figure 3). Figure 3 also shows these results of the other non-surgical treatments for hip OA patients and the same results for knee OA patients.

Though there were differences in percentages between satisfied hip and knee OA patients of up to 32% for instance for the effect of tramadol on pain, no significant differences were found for the distribution of satisfaction rates between hip and knee OA patients.

### Sensitivity analyses

Of the 175 patients, 112 (64%) completed the questionnaire preoperatively. No statistically significant differences were found in baseline characteristics except for country region: 54% of preoperative patients came from the south versus 84% of postoperative. No statistically significant differences were found in received treatments except for education in treatments of OA, which was received by 74% of the preoperative patients and 57% of postoperative (p = 0.03). Patients on the waiting list for surgery were less often satisfied than patients already operated on regarding physical therapy for stiffness (44% versus 61%, p = 0.03), NSAIDs for swelling (40% versus 65%, p = 0.04), NSAIDs for sport (29% versus 69%, p = 0.01), and tramadol for pain (38% versus 67%, p = 0.05).

Of the 175 patients, 57 (33%) had paid employment at the time of surgery. Patients with paid employment were younger (59 years, SD = 7) than patients without paid employment (69 years, SD = 7; p < 0.001) and they were more highly educated than patients without paid employment (p < 0.01). In addition, diabetes mellitus was less common among patients with paid employment (p = 0.04) and rheumatic diseases were more common (p = 0.01). Lifestyle advice and exercise-based therapy were received by more hip and knee OA patients with paid employment (both 77%) compared with patients without paid employment (52% and 54%, respectively; p < 0.01). Hip and knee OA patients with paid employment were more satisfied with the effect of NSAIDs for work participation (56% versus 33%; p = 0.02), intra-articular injection (54% versus 20%; p < 0.01) and exercise-based therapy (42% versus 40%; p = 0.6) than patients without paid employment at the time of surgery.

## Discussion

The most important finding of the present study was an underuse of non-surgical treatments reported by hip and knee OA patients. In terms of the SCS, step 1 treatments were received by up to 79%, step 2 by up to 66%, and step 3 by up to 47%. Regarding satisfaction with treatments, more than half of the patients were satisfied with the effect on pain of acetaminophen (hip), NSAIDs (hip and knee), tramadol (knee), exercise-based therapy (hip and knee), and intra-articular injection (knee). However, satisfaction rates with non-surgical treatments for work and sports/leisure participation were generally low.

The comparison of non-surgical treatment utilization over time revealed that no treatment was received more often than in 2013 in the Netherlands. Non-surgical treatment remains underutilized and this is especially true for treatments of steps 2 and 3. These results are in line with recent findings from Denmark showing underuse of non-surgical knee OA treatment before referral to an orthopedic surgeon (Ingelsrud et al. 2020). Underuse of NSAIDs and intra-articular injection might be related to safety implications (Charlesworth et al. 2019). However, it seems unlikely that this explains why these treatments are not used or received by more than 50% of patients given the recommendation in the SCS strategy. This assumption is also supported by the positive results of hyaluronic acid on knee-related function and knee complaints according to a recent RCT among working-age knee OA patients (Hermans et al. 2019). Also, our sensitivity analyses showed that patients with paid employment were especially satisfied with the effects of these intra-articular injections on work participation. The same was true for NSAIDs. Given the possible positive effect of these treatments on work participation for half of the patients, these treatments might be underutilized for working-age patients. Unfortunately, no work-related outcomes are presented by Hermans et al. (2019).

Several reasons for the underuse of the non-surgical treatments can be mentioned. For instance, Hofstede et al. (2016) showed that an important barrier might be that orthopedic surgeons have little faith in the effectiveness of these treatments. The same might be true for general practitioners’ attitudes towards non-surgical treatment (Smink et al. 2014). For patients, positive experiences with surgery of people in their own environment may lead to the belief that non-surgical therapy is inferior to surgery (Hofstede et al. 2016), even though about 10% of hip OA patients and 20% of knee OA patients reported persistent pain after joint replacement, even after generic or indicated pain education (Beswick et al. 2012, Louw et al. 2019, Birch et al. 2020). Consequently, better education on the effectiveness of non-surgical treatments for both patients and healthcare professionals might support non-surgical treatment utilization. Indeed, active regional implementation of the SCS, including a patient education booklet, educational outreach visits, and providing reminder material to general practitioners, resulted in increased adherence to the SCS (Smink et al. 2014). These findings justify further dissemination of this implementation strategy to healthcare providers of hip and knee OA patients. Our comparison of non-surgical treatment utilization over time also showed that initiatives based on the Choosing Wisely campaign will not produce the desired results when no additional implementation measures, as stated above, are taken.

That patients were older and complaints lasted longer in the present study compared with the study from 2013 (Hofstede et al. 2015) can be interpreted as a trend towards a “wait and see” policy before arthroplasty. If this is the case, this waiting time can be used more effectively by non-surgical treatment. For instance, being overweight is more common in the present study and diet therapy is used in only a third or less by the responding obese or overweight patients. The fact that at least 30% of patients are satisfied with the effect of diet therapy on pain, and in knee OA patients also on stiffness, ADL, and sport/leisure, may support better use of this treatment.

In this study, the core outcome domain set for total joint replacement (TJR) (Singh et al. 2017) recommending the use of pain, satisfaction, and participation is used to align outcomes of received non-surgical treatments with the outcomes of TJR. These outcomes are essential for providing better patient-tailored care, particularly for chronic diseases like OA (Karsdal et al. 2014, Witjes et al. 2017). For example, work-disabled knee OA patients may be advised that hyaluronic acid in combination with exercise-based therapy could provide the optimal non-surgical treatment for pain and work participation (Hermans et al. 2019). Additionally, patients might be encouraged to discuss which non-surgical treatments might be most beneficial for their specific needs regarding symptoms and participation. Lastly, data on patient satisfaction regarding the effects on symptoms and participation of non-surgical treatment might stimulate healthcare professionals and researchers to prescribe and develop more effective treatment combinations. While interest in and social demand for these non-surgical treatments is growing (Karsdal et al. 2014, Skou et al. 2018), we hope that an increasing number of hip and knee OA patients receive non-surgical treatment, thereby enabling surgery to be postponed.

### Strengths and limitations

Our study has some limitations. Most importantly, we performed a retrospective study relying on self-reported data, thus making our findings prone to recall bias. Therefore, in line with previous recommendations, we limited inclusion to patients who were scheduled for total hip or total knee arthroplasty or who underwent total hip or total knee arthroplasty no longer than 6 months previously (Hofstede et al. 2015). Another limitation might be that the patients’ satisfaction rates with the treatment effects, given the indication for surgery, are probably lower than the rates in patients in an earlier stage of OA. Thus, regarding external validity of our findings, our satisfaction rates may only be applicable to preoperative hip and knee OA patients.

A strength of our study is that for the first time, as far as we are aware, we evaluated patient satisfaction regarding the effects of non-surgical treatments in hip and knee OA not only on pain but also on other symptoms like stiffness, and on participation as in work. Moreover, we were able to make a comparison regarding utilization of non-surgical treatment between the present study and data collected in a similar manner in 2013 (Hofstede et al. 2015). Another strength is our evaluation after the implementation of an evidence-based non-surgical SCS and the Choosing Wisely campaign. Therefore, our findings on utilization of non-surgical therapy may be valid for countries with comparable initiatives and healthcare provision, although financial compensation for and social acceptance of treatments may differ according to country.

## Conclusion

Non-surgical treatments for hip and knee OA patients appear underutilized in the Netherlands. Of the patients referred for arthroplasty, generally more were satisfied with the effect of non-surgical treatment on pain than with the effect on participation in work and sports/leisure. Better insight into patients’ satisfaction regarding non-surgical treatment effects on symptoms and participation might stimulate patient-centered care and thereby increase better adherence.

This study was set up by a joint effort of AH, KK, LB, GK, SW, TB, RG, and PK. SW, AH, RG, and PK conceived and designed the study. AH designed and distributed the surveys and completed the data collection. YZ and PK were responsible for the processing and analyses of data. YZ drafted the first version of the article. AH and PK contributed to the further drafting of the article. KK, LB, GK, SW, TB, and RG all agreed on conception and design and contributed equally to interpretation of data and its critical revision. YZ and AH edited the manuscript and YZ wrote the final draft. PK is guarantor of the article. Final approval of the version for publication was agreed upon by all authors.

The authors would like to thank all patients who participated in this study.

Patient characteristics of the total group of hip and knee OA patients and each group separately. Values are number (%) unless otherwise specified

**Table:** 

	Total OA	Hip OA **^a^**	Knee OA **^a^**
	group	n = 82	n = 92
Variable	n = 175	(47%)	(53%)
Mean age (SD) **^b^**	66 (8)	66 (9)	65 (7)
Female sex, n (%) **^c^**	100 (57)	42 (51)	57 (62)
BMI, median [IQR] **^d, e^**	29 [25–33]	28 [24–31]	31 [27–36]
Educational level **^d^**			
Primary	48 (27)	19 (23)	29 (32)
Secondary	72 (41)	35 (43)	36 (39)
College/university	55 (31)	28 (34)	27 (29)
Comorbidity **^d, f^**			
Diabetes mellitus	19 (11)	6 (7)	13 (14)
Cerebrovascular accident	10 (6)	3 (4)	7 (8)
Cancer	12 (7)	5 (6)	7 (8)
Cardiac diseases	20 (11)	5 (6)	15 (16)
Migraine/severe headache	9 (5)	5 (6)	4 (4)
High blood pressure	54 (31)	23 (28)	31 (34)
Lung diseases	20 (11)	6 (7)	14 (15)
Rheumatic diseases	20 (11)	8 (10)	12 (13)
Other	22 (13)	9 (11)	13 (14)
Work circumstances **^d^**			
Paid employment	47 (27)	22 (27)	25 (27)
Self-employed	10 (6)	5 (6)	5 (5)
No paid employment **^g^**	118 (67)	55 (67)	62 (67)
Onset of OA complaints **^d, e^**			
< 1 year	9 (5)	4 (5)	5 (5)
1–5 years	78 (45)	48 (59)	30 (33)
> 5 years	28 (16)	12 (15)	15 (16)
> 10 years	37 (21)	12 (15)	25 (27)
> 20 years	23 (13)	6 (7)	17 (19)
Region of the country **^d^**			
North	37 (21)	21 (26)	16 (17)
Middle	25 (14)	12 (15)	12 (13)
South	113 (65)	49 (60)	64 (70)

**^a^** 1 hip or knee is missing; IQR = interquartile range; SD = standard deviation;

**^b^** Student’s t-test;

**^c^** Fisher–Freeman–Halton exact test;

**^d^** Mann–Whitney U-test;

**^e^**Significant difference between groups p ≤ 0.05;

**^f^** counting > 100% while more than one answer possible.

**^g^**Unemployed, retirement, etc.

## References

[CIT0001] Bernstein J. Not the last word: choosing wisely. Clin Orthop Relat Res 2015; 473(10): 3091–7. 26267312 10.1007/s11999-015-4490-8 PMC4562936

[CIT0002] Berwick D M, Hackbarth A D. Eliminating waste in US health care. JAMA 2012; 307(14): 1513–16. 22419800 10.1001/jama.2012.362

[CIT0003] Beswick A D, Wylde V, Gooberman-Hill R, Blom A, Dieppe P. What proportion of patients report long-term pain after total hip or knee replacement for osteoarthritis? A systematic review of prospective studies in unselected patients. BMJ Open 2012; 2(1): e000435. 10.1136/bmjopen-2011-000435 PMC3289991 22357571

[CIT0004] Birch S, Stilling M, Mechlenburg I, Hansen T B. No effect of cognitive behavioral patient education for patients with pain catastrophizing before total knee arthroplasty: a randomized controlled trial. Acta Orthop 2020; 91(1): 98–103 31762342 10.1080/17453674.2019.1694312 PMC7006640

[CIT0005] Charlesworth J, Fitzpatrick J, Perera N K P, Orchard J. Osteoarthritis—a systematic review of long-term safety implications for osteoarthritis of the knee. BMC Musculoskelet Disord 2019; 20(1): 151. 30961569 10.1186/s12891-019-2525-0 PMC6454763

[CIT0006] Culliford D, Maskell J, Judge A, Cooper C, Prieto-Alhambra D, Arden N K. Future projections of total hip and knee arthroplasty in the UK: results from the UK Clinical Practice Research Datalink. Osteoarthritis Cartilage 2015; 23(4): 594–600. 25579802 10.1016/j.joca.2014.12.022

[CIT0007] Grimshaw J, Eccles M, Tetroe J. Implementing clinical guidelines: current evidence and future implications. J Contin Educ Health Prof 2004; 24(Suppl. 1): S31–S7. 15712775 10.1002/chp.1340240506

[CIT0008] Hermans J, Bierma-Zeinstra S M A, Bos P K, Niesten D D, Verhaar J A N, Reijman M. The effectiveness of high molecular weight hyaluronic acid for knee osteoarthritis in patients in the working age: a randomised controlled trial. BMC Musculoskelet Disord 2019; 20(1): 196. 31064359 10.1186/s12891-019-2546-8 PMC6503549

[CIT0009] Hofstede S N, Vliet Vlieland T P, van den Ende C H, Nelissen R G, Marang-van de Mheen P J, van Bodegom-Vos L. Variation in use of non-surgical treatments among osteoarthritis patients in orthopaedic practice in the Netherlands. BMJ Open 2015; 5(9): e009117. 10.1136/bmjopen-2015-009117 PMC4567674 26353874

[CIT0010] Hofstede S N, Marang-van de Mheen P J, Vliet Vlieland T P, van den Ende C H, Nelissen R G, van Bodegom-Vos L. Barriers and facilitators associated with non-surgical treatment use for osteoarthritis patients in orthopaedic practice. PLoS One 2016; 11(1): e0147406. 26799974 10.1371/journal.pone.0147406 PMC4723077

[CIT0011] Ingelsrud L H, Roos E M, Gromov K, Jensen S S, Troelsen A. Patients report inferior quality of care for knee osteoarthritis prior to assessment for knee replacement surgery: a cross-sectional study of 517 patients in Denmark. Acta Orthop 2020; 91(1): 82–7. 31635504 10.1080/17453674.2019.1680180 PMC7006715

[CIT0012] Karsdal M A, Christiansen C, Ladel C, Henriksen K, Kraus V B, Bay-Jensen A C. Osteoarthritis: a case for personalized health care? Osteoarthritis Cartilage 2014; 22(1): 7–16. 24216058 10.1016/j.joca.2013.10.018

[CIT0013] Kuijer P P, Kievit A J, Pahlplatz T M, Hooiveld T, Hoozemans M J, Blankevoort L, Schafroth M U, van Geenen R C, Frings-Dresen M H. Which patients do not return to work after total knee arthroplasty? Rheumatol Int 2016; 36(9): 1249–54. 27342661 10.1007/s00296-016-3512-5 PMC4983277

[CIT0014] Kurtz S M, Lau E, Ong K, Zhao K, Kelly M, Bozic K J. Future young patient demand for primary and revision joint replacement: national projections from 2010 to 2030. Clin Orthop Relat Res 2009; 467(10): 2606–12. 19360453 10.1007/s11999-009-0834-6 PMC2745453

[CIT0015] Louw A, Puentedura E J, Reed J, Zimney K, Grimm D, Landers M R. A controlled clinical trial of preoperative pain neuroscience education for patients about to undergo total knee arthroplasty. Clin Rehabil 2019; 33(11): 1722–31. 31213078 10.1177/0269215519857782

[CIT0016] McAlindon T E, Bannuru R R, Sullivan M C, Arden N K, Berenbaum F, Bierma-Zeinstra S M, Hawker G A, Henrotin Y, Hunter D J, Kawaguchi H, Kwoh K, Lohmander S, Rannou F, Roos E M, Underwood M. OARSI guidelines for the non-surgical management of knee osteoarthritis. Osteoarthritis Cartilage 2014; 22(3): 363–88. 24462672 10.1016/j.joca.2014.01.003

[CIT0017] NOV. Choosing wisely in orthopedics; Verstandige keuzes binnen de orthopedie; 1. geen heup- en knievervanging zonder adequaat conservatieve behandeling. In: Verstandig kiezen. ZonMw (The Netherlands Organisation for Health Research and Development) NOV NPCF; 2015.

[CIT0018] Otten R, van Roermund P M, Picavet H S. [Trends in the number of knee and hip arthroplasties: considerably more knee and hip prostheses due to osteoarthritis in 2030]. Ned Tijdschr Geneeskd 2010; 154: A1534. 20619009

[CIT0019] Singh J A, Dowsey M M, Dohm M, Goodman S M, Leong A L, Scholte Voshaar M, Choong P F. Achieving consensus on total joint replacement trial outcome reporting using the OMERACT filter: endorsement of the final core domain set for total hip and total knee replacement trials for endstage arthritis. J Rheumatol 2017; 44(11): 1723–6. 28089984 10.3899/jrheum.161113

[CIT0020] Skou S T, Roos E M, Laursen M B, Rathleff M S, Arendt-Nielsen L, Rasmussen S, Simonsen O. Total knee replacement and non-surgical treatment of knee osteoarthritis: 2-year outcome from two parallel randomized controlled trials. Osteoarthritis Cartilage 2018; 26(9): 1170–80. 29723634 10.1016/j.joca.2018.04.014

[CIT0021] Smink A J, van den Ende C H, Vliet Vlieland T P, Swierstra B A, Kortland J H, Bijlsma J W, Voorn T B, Schers H J, Bierma-Zeinstra S M, Dekker J. “Beating osteoarthritis”: development of a stepped care strategy to optimize utilization and timing of non-surgical treatment modalities for patients with hip or knee osteoarthritis. Clin Rheumatol 2011; 30(12): 1623–9. 21887488 10.1007/s10067-011-1835-x

[CIT0022] Smink A J, Bierma-Zeinstra S M, Schers H J, Swierstra B A, Kortland J H, Bijlsma J W, Teerenstra S, Voorn T B, Dekker J, Vliet Vlieland T P, van den Ende C H. Non-surgical care in patients with hip or knee osteoarthritis is modestly consistent with a stepped care strategy after its implementation. Int J Qual Health Care 2014; 26(4): 490–8. 24845068 10.1093/intqhc/mzu058

[CIT0023] Smith T O, Hawker G A, Hunter D J, March L M, Boers M, Shea B J, Christensen R, Guillemin F, Terwee C B, Williamson P R, Dodd S, Roos E M, Loeser R F, Schnitzer T J, Kloppenburg M, Neogi T, Ladel C H, Kalsi G, Kaiser U, Buttel T W, Ashford A E, Mobasheri A, Arden N K, Tennant A, Hochberg M C, de Wit M, Tugwell P, Conaghan P G. The OMERACT-OARSI core domain set for measurement in clinical trials of hip and/or knee osteoarthritis. J Rheumatol 2019; 46(8): 981–9. 30647185 10.3899/jrheum.181194 PMC10753652

[CIT0024] Snijders G F, den Broeder A A, van Riel P L, Straten V H, de Man F H, van den Hoogen F H, van den Ende C H, Group N S. Evidence-based tailored conservative treatment of knee and hip osteoarthritis: between knowing and doing. Scand J Rheumatol 2011; 40(3): 225–31. 21261551 10.3109/03009742.2010.530611

[CIT0025] Stigmar K, Dahlberg L E, Zhou C, Jacobson Lidgren H, Petersson I F, Englund M. Sick leave in Sweden before and after total joint replacement in hip and knee osteoarthritis patients. Acta Orthop 2017; 88(2): 152–7. 27996342 10.1080/17453674.2016.1269051 PMC5385109

[CIT0026] Witjes S, van Geenen R C, Koenraadt K L, van der Hart C P, Blankevoort L, Kerkhoffs G M, Kuijer P P. Expectations of younger patients concerning activities after knee arthroplasty: are we asking the right questions? Qual Life Res 2017; 26(2): 403–17. 27492606 10.1007/s11136-016-1380-9 PMC5288419

[CIT0027] Zhang W, Nuki G, Moskowitz R W, Abramson S, Altman R D, Arden N K, Bierma-Zeinstra S, Brandt K D, Croft P, Doherty M, Dougados M, Hochberg M, Hunter D J, Kwoh K, Lohmander L S, Tugwell P. OARSI recommendations for the management of hip and knee osteoarthritis, part III: Changes in evidence following systematic cumulative update of research published through January 2009. Osteoarthritis Cartilage 2010; 18(4): 476–99. 20170770 10.1016/j.joca.2010.01.013

